# Detection of translational noncrystallographic symmetry in Patterson functions

**DOI:** 10.1107/S2059798320016836

**Published:** 2021-02-05

**Authors:** Iracema Caballero, Massimo D. Sammito, Pavel V. Afonine, Isabel Usón, Randy J. Read, Airlie J. McCoy

**Affiliations:** a Crystallographic Methods, Institute of Molecular Biology of Barcelona (IBMB–CSIC), Baldiri Reixac 15, 08028 Barcelona, Spain; b Department of Haematology, Cambridge Institute for Medical Research, University of Cambridge, Hills Road, Cambridge CB2 0XY, United Kingdom; c Lawrence Berkeley National Laboratory, One Cyclotron Road, BLDG 64R0121, Berkeley, CA 93720, USA; d ICREA, Passeig de Lluís Companys 23, 08010 Barcelona, Spain

**Keywords:** translational noncrystallographic symmetry, maximum likelihood, intensity statistics, molecular replacement

## Abstract

Detection of translational noncrystallographic symmetry (TNCS) can be critical for success in crystallographic phasing, particularly when molecular-replacement models are poor or anomalous phasing information is weak. If the correct TNCS is detected then expected intensity factors for each reflection can be refined, so that the maximum-likelihood functions underlying molecular replacement and single-wavelength anomalous dispersion use appropriate structure-factor normalization and variance terms. Here, an analysis of a curated database of protein structures from the Protein Data Bank to investigate how TNCS manifests in the Patterson function is described. These studies informed an algorithm for the detection of TNCS, which includes a method for detecting the number of vectors involved in any commensurate modulation (the TNCS order). The algorithm generates a ranked list of possible TNCS associations in the asymmetric unit for exploration during structure solution.

## Abstract 2

Translational noncrystallographic symmetry (TNCS) was analysed using a curated database of 80 000 protein structures to inform an algorithm for the detection of TNCS order.

## 1. Introduction   

Translational noncrystallographic symmetry (TNCS) arises when the asymmetric unit contains components that are oriented in (nearly) the same way and can be superimposed by a translation that does not correspond to any symmetry operation in the space group. There is overall modulation of the intensities: systematically strong and systematically weak intensities (Chook *et al.*, 1998 ▸). Structure determination and refinement is problematic if the systematic modulation is not taken into account, because the intensity modulation caused by TNCS breaks the implicit assumptions used in likelihood-based methods that the intensities, and the errors in predicting the intensities from the model, follow an isotropic Wilson distribution (Wilson, 1949 ▸).

**Figure 1:**
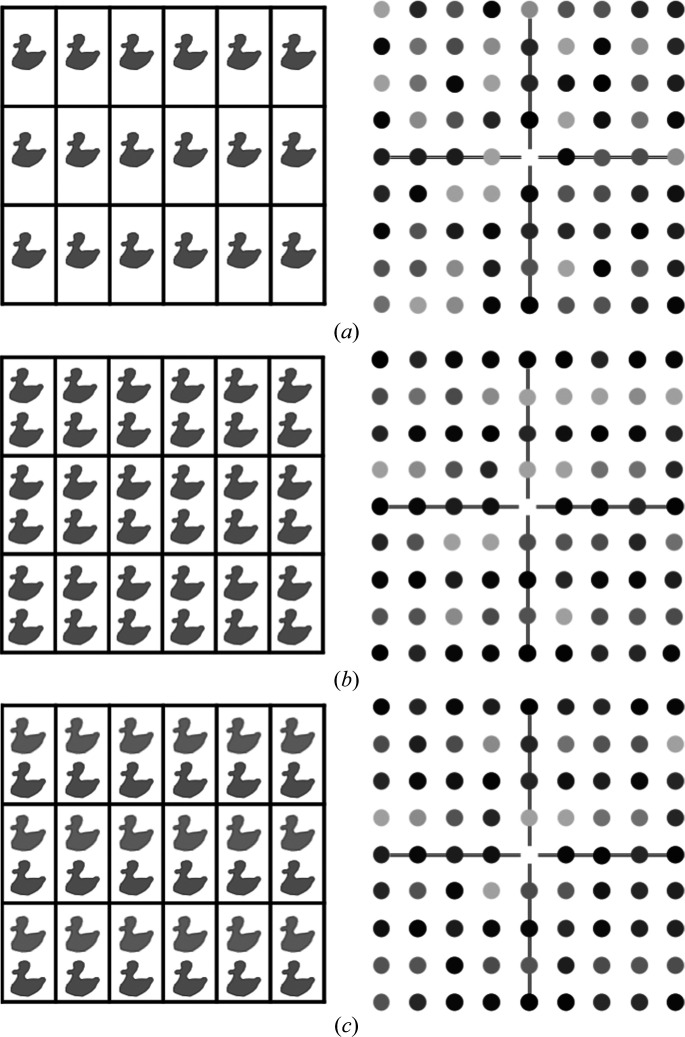
Modulation of diffraction intensities for a molecule (represented by a duck) with significant anomalous scattering, so that Friedel’s law is not obeyed. The arrangement of molecules in the crystal is shown with the *y* axis vertical (left) and the intensities shown on a square grid for the *h*0*l* layer of reciprocal space (right). (*a*) A crystal without TNCS and intensities with no modulation. (*b*) A crystal with TNCS between two molecules, shifted by a vector close to half the *y*-axis lattice translation. The intensities show weaker than average intensity reflections in the odd rows and stronger than average intensities in the even rows. (*c*) A crystal with TNCS between two molecules, shifted by a vector close to half the vertical lattice translation and with a 20° rotation. The intensities show the same pattern of intensity modulations as in (*b*), but not as pronounced.

The modulations of the intensities arise because the contribution to a structure factor of molecules related by TNCS have the same (or similar) amplitudes but have relative phases determined by the projection of the translation vector on the diffraction vector. As a result, they interfere constructively for some reflections and destructively for others, so that there is a systematic modulation of the sum of their contributions. The planes affected by intensity modulation are perpendicular to the translation vectors between copies related by TNCS (TNCS vectors). The degree of modulation is less significant if there are rotational and/or conformational differences between the copies, and decreases with increasing resolution. For this reason, in addition to the TNCS vector it is also necessary to estimate any small rotational differences in their orientations (TNCS rotations) and the size of random coordinate differences (TNCS r.m.s.d.) caused by conformational differences (Read *et al.*, 2013 ▸) in order to correctly account for TNCS modulation (Fig. 1 ▸).

The parameters characterizing TNCS (TNCS vector, TNCS rotation and TNCS r.m.s.d.) are used to generate expected intensity factors for each reflection. Note that the total expected intensity factor for a reflection includes the usual integer factor for the number of times the Miller index of a reflection is identical under all of the distinct pure rotational symmetry operations of the space group (Stewart & Karle, 1976 ▸). The TNCS component of the expected intensity factor that models the modulations observed in the data is non-integer (Read *et al.*, 2013 ▸), being below 1 for the systematically weak reflections and above 1 for the systematically strong reflections.

After initial estimation, the parameters of the TNCS model are refined, via the expected intensity factors for each reflection derived from the TNCS model, using a likelihood function given by the Wilson distribution of the data (McCoy, 2007 ▸).

TNCS does not necessarily associate two components in the asymmetric unit, but may relate three or more (*n*) components associated by a series of vectors that are multiples of 1, 2, 3 … (*n* − 1) times a basic translation vector. We call *n* the order of the TNCS and indicate it as TNCS_*n*_. Where *n* times the basic translation vector equates to (or is very close to) a sum of integer multiples of the unit-cell basis vectors, the TNCS describes a pseudo-cell, and this case is known as commensurate modulation.

The presence of TNCS is shown by the presence of a strong off-origin peak in the Patterson function (Patterson, 1935 ▸) caused by the overlap of multiple parallel and equal-length interatomic vectors. In *phenix.xtriage* (Zwart *et al.*, 2005 ▸), TNCS is flagged as present if a Patterson function calculated with data from 5 to 10 Å resolution has a peak more than 15 Å from the origin which is at least 20% of the origin-peak height. The rationale for the resolution limits is to enhance the signal for the low-resolution molecular transform, and the rationale for the distance threshold is to exclude the Patterson function origin peak and any internal pseudo-translational symmetry such as in helices. However, there has not been a systematic study of the parameters of this approach, nor of how accurate it is in the detection of TNCS. In addition, this approach does not automatically give the order of the TNCS, which is critical for correcting the modulations. In the context of developing automated structure-solution strategies, we are also interested in ranking alternative hypotheses for TNCS.

## 2. Materials and methods   

### 2.1. Database   

The database for the study was derived from an initial subset of 90 083 crystal structures from the PDB (Burley *et al.*, 2019 ▸) deposited between 1976 and 2018 and for which there were also deposited X-ray intensities or amplitudes. Structures containing nucleic acids or highly α-helical proteins (75% or more helical content), such as coiled coils, were excluded, since these structural classes are known to have characteristically high intensity modulation even in the absence of TNCS. The helical content was calculated following the distribution of characteristic vectors (CVs; Medina *et al.*, 2020 ▸) defined by the centroids of C^α^ and carbonyl O atoms from consecutive and overlapping heptapeptides. The intensity modulations generated by the helical repeats in these structures cannot be corrected by modelling them as TNCS-generated modulations and thus are beyond the scope of this study. Also excluded from the database were collagens, viruses, small nonproteins (antibiotics and peptides), structures with a mean occupancy of less than 0.75 and structures where only the C^α^-atom coordinates were deposited.

Curation included the following checks on data quality: (i) retracted entries were deleted, (ii) obsolete structures were replaced by the valid entries as of October 2018, (iii) where PDB entries had MTRIX cards to represent NCS operators, the *phenix.pdb.mtrix_reconstruction* script (Liebschner *et al.*, 2019 ▸) was used to reconstruct the crystallographic asymmetric unit and the transformation given in the SCALE cards was used to place the model in the asymmetric unit, and (iv) data in the form of unmerged intensities were converted to merged intensities with *phenix.reflection_file_converter* using the --non-anomalous option (Liebschner *et al.*, 2019 ▸). Finally, a small subset of structures for which our scripts failed were substituted with data or coordinates from the PDB-REDO database (Joosten *et al.*, 2012 ▸) if that solved the issue, or else were deleted without further examination of the causes.

Since the TNCS modulations of intensities become less pronounced at high resolution, where the data extended to high resolution they were truncated to 3 Å resolution in order to save run time in the calculations. Our initial studies were performed without regard to the completeness of the data, but we observed that incomplete data caused outliers in our preliminary analysis, and so our primary database was further curated to remove cases where the data were less than 80% complete, and a separate database was maintained to further study the effects of incompleteness.

The final curated database contains 80 482 structures. Its characteristics and genesis are summarized in Table 1 ▸. The small database of structures with data completeness less than 80% consisted of 1294 cases. Both databases are available upon request from the authors.

### 2.2. Computing and software   

The atomic coordinates of structures deposited in the PDB were analysed and TNCS, if any, was identified using the *mmtbx.ncs* package from the *mmtbx* module of the *Computational Crystallography Toolbox* (*cctbx*; Grosse-Kunstleve *et al.*, 2002 ▸). In this algorithm, chains with high sequence identity are identified. They are then structurally superimposed, testing each crystal symmetry operation including the identity, and if they superimpose with a translation then the pair is added to a growing list of TNCS-related chains in the asymmetric unit. The translation can include a rotational tolerance defined by an angular threshold. After all combinations of sequence-matched chains and symmetry operations have been considered, the list is analysed to find the largest TNCS order. Importantly, the analysis forces the TNCS-related molecules to form a closed group; so, for example, if the rotational tolerance is 3°, and *A* superimposes on *B* with a 2° rotation, *B* superimposes on *C* with a 2° rotation and *A* superimposes on *C* with a 4° rotation, then *A*, *B* and *C* form a TNCS group of order 3 even though *A* and *C* do not superimpose within the tolerance of 3°. In the limit of high angular tolerances, high-order rotational symmetry will be misidentified as high-order translational symmetry (see, for example, Albertini *et al.*, 2006 ▸; PDB entry 2gtt). The package reports the chain identifier of the TNCS-related chains, the TNCS vector in fractional and orthogonal coordinates, the rotational difference and the percentage of total scattering for the pairs of molecules related by TNCS.

The Patterson function was calculated from the deposited data. Where mean intensities were available, reflections recorded as net positive were used for the calculation. If only anomalous intensities were available, a mean intensity was calculated as a simple average of the Friedel mates or using the singleton intensity if only one Friedel mate was present. If only structure-factor amplitudes were available and these had been generated by the French and Wilson procedure (French & Wilson, 1978 ▸) then the transformation was reversed to obtain intensities (Read & McCoy, 2016 ▸). If only structure-factor amplitudes were available and these had not been generated using the French and Wilson algorithm, the intensity was taken as the square of the structure-factor amplitude; the information loss meant that reflections with negative experimental intensity were set to zero intensity. All data were used without applying an *I*/σ(*I*) selection criterion.

The TNCS correction terms were calculated with the *Phasertng* software package (McCoy *et al.*, 2021 ▸) using algorithms like those implemented in *Phaser* (McCoy, 2007 ▸; Read *et al.*, 2013 ▸; Sliwiak *et al.*, 2014 ▸; Read & McCoy, 2016 ▸; Jamshidiha *et al.*, 2019 ▸). When the TNCS order is greater than 2 the relative orientations between the components related by the TNCS are not included in the model for TNCS, but their effect is absorbed approximately by the TNCS r.m.s.d. parameter. Correction terms are applied to the observed and calculated structure factors during all likelihood calculations involved in molecular replacement and single anomalous dispersion (SAD) phasing.

Figures were prepared with the *PyMOL* Molecular Graphics System (version 1.8; Schrödinger) and *Matplotlib* version 1.5.3 (Hunter, 2007 ▸).

The decision tree was generated using the scikit-learn Python library version 0.18.1 (Pedregosa *et al.*, 2011 ▸).

Calculations were performed on a multiprocessing workstation with two quad-core Intel Xeon processors X5560 at 2.80 GHz with 24 GB RAM and on an 18-core workstation with Intel Core i9-9980XE at 3.00 GHz with 64 GB RAM, both with the operating system Debian GNU/Linux 9.

## 3. Results   

### 3.1. TNCS in real space   

The first question to arise when studying TNCS is ‘What constitutes TNCS?’ This is not a simple question to answer. The effects of TNCS form a continuum between exact TNCS and molecules in the asymmetric unit oriented with large rotation angles with respect to one another (general NCS).

Our initial approach was to use the coordinates for decision making. Whether or not coordinates have TNCS depends on the choice of a rotational tolerance. In our experience of TNCS parameter refinement, TNCS rotations can refine to values up to 10° (Read *et al.*, 2013 ▸). Coordinate analysis was therefore carried out exploring a wide range of rotational tolerances from 0° to 20°. The results are shown in Table 2 ▸. At small angular tolerances of less than 5°, one in 20 of the structures in the database were flagged as having TNCS, while at 10° tolerance this had increased to nearly one in ten and by 20° it was one in seven. Furthermore, in some cases the order of the TNCS also increased with tolerance; 6% of the TNCS was higher order TNCS (*n* > 2) at 2° tolerance and 14% at 20° tolerance. Most of the increase in the order of the TNCS occurred when increasing the tolerance from 2° to 5°, because higher order TNCS often has subsets of components that are more closely related than others, and what appears to be complex low-order TNCS at small tolerances reduces to a simple high-order TNCS at larger tolerances. We refer to the coordinates-based test for TNCS as pdb-TNCS(*r*°), where the angle *r* is the angular tolerance and the value is true/false.

### 3.2. Patterson function vector-length threshold   

Patterson function intramolecular vectors cluster around the Patterson function origin peak. These peaks, which constitute noise in the context of searching for TNCS vectors, can be excluded by setting a minimum vector-length threshold. The shortest TNCS vector that is possible in any given case will depend on the shortest intermolecular spacing, and this distance could be used as a constraint on the TNCS vector. However, the shortest extent is not known before structure determination; only by assuming a spherical molecule could a reasonable estimate of the average molecular extent be made from the molecular weight for a completely unknown structure. Independently, there is a need to exclude short vectors because of pseudo-symmetry in secondary-structure elements, such as α-helices and β-sheets. The distances arising from these pseudo-symmetries are less than 15 Å, which has been used as the threshold distance for exclusion (Zwart *et al.*, 2005 ▸, 2008 ▸). We wished to determine whether this distance was larger than any TNCS vector in the PDB.

**Figure 2:**
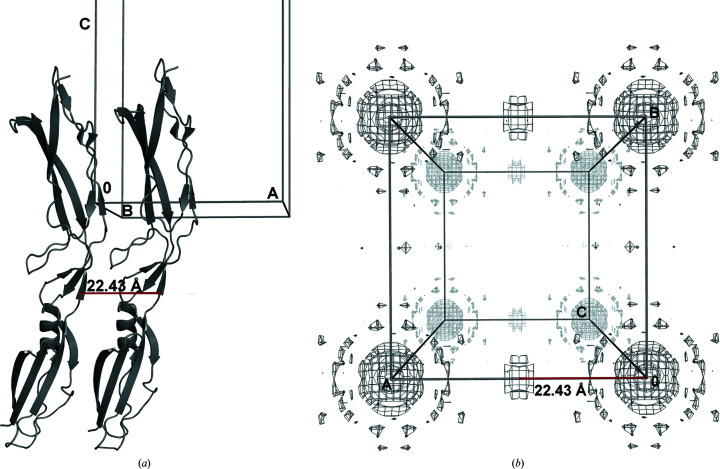
(*a*) TNCS-related molecules of PDB entry 3i57. (*b*) Patterson function map of PDB entry 3i57, drawn in 3D perspective projection, showing the origin peaks and the peak 22.43 Å from the origin which corresponds to the TNCS translation (0.5, 0.0, 0).

The shortest TNCS vector in our database was 22.43 Å for the structure with PDB code 3i57 (MacKenzie *et al.*, 2009 ▸), with a fractional translation vector of (0.5, 0, 0) and a rotational tolerance of 6.7°. The structure of PDB entry 3i57 is shown in Fig. 2 ▸(*a*) and its Patterson function in Fig. 2 ▸(*b*). We conclude that the 15 Å distance from the origin of the Patterson function peak is suitable for excluding self-vectors while not excluding any true TNCS vectors.

### 3.3. Patterson function peak threshold   

**Figure 3:**
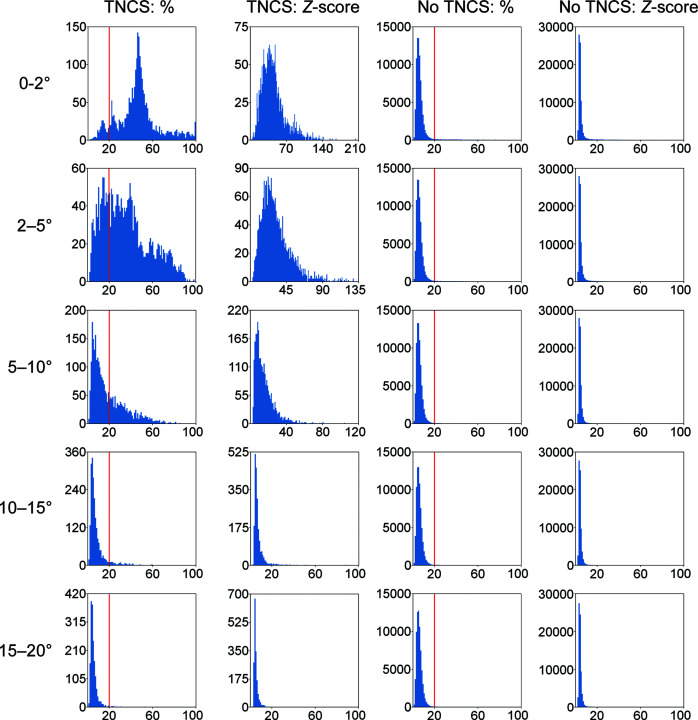
Noncumulative histograms of the number of structures with different values for the highest non-origin peak, depending on rotational tolerances. The Patterson function was calculated with data from 5 to 10 Å resolution; graphs for other Patterson function resolution ranges are provided in the supporting information. The first and second columns are for cases with TNCS and the third and fourth columns for cases without TNCS; the first and third columns express the maximal non-origin peak height as a percentage of the origin peak height, while the second and fourth columns express it as a *Z*-score. A red line is shown at Patterson-20%, which is the previous threshold for determining the presence of TNCS.

Our next step was to investigate the correlation of pdb-TNCS(*r*°) with the peak heights in the Patterson function. Fig. 3 ▸ shows the histograms for the distribution of top non-origin Patterson function peak heights. Results are shown for Patterson functions calculated with data between 5 and 10 Å resolution and with different pdb-TNCS(*r*°) angular tolerances. Other resolution ranges are shown in Supplementary Fig. S1. The top non-origin peak was expressed as a percentage of the height of the Patterson function origin peak and as a *Z*-score value (the number of standard deviations above the mean value). For pdb-TNCS(2°), the histogram showed that the traditional Patterson 20% of the origin peak threshold was broadly correct; this gave an accuracy (defined below) of 96%. However, for pdb-TNCS(15°) the accuracy began to break down (94%), and by pdb-TNCS(20°) it was only 92%.

### 3.4. Decision tree   

**Figure 4:**
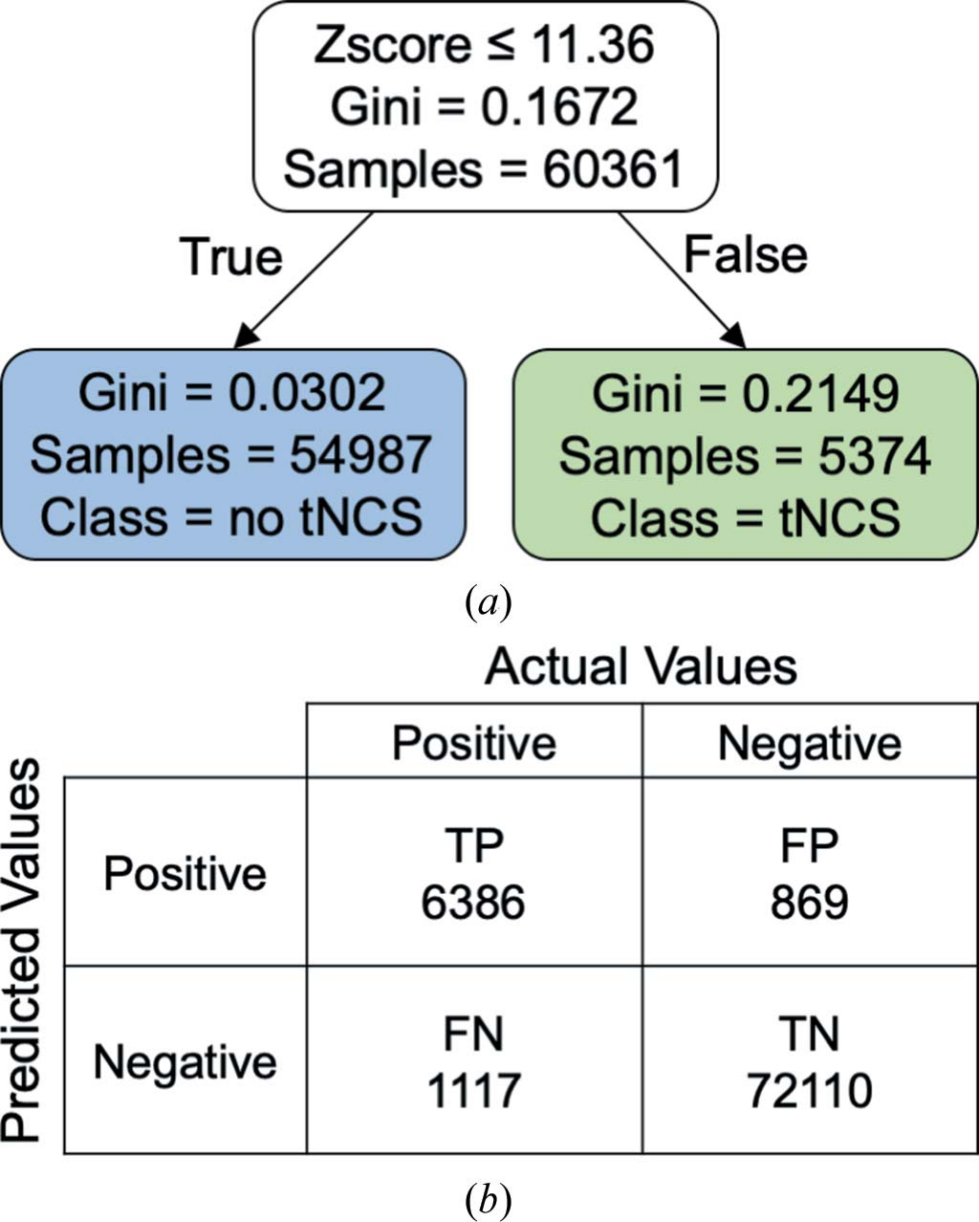
(*a*) Decision tree for pdb-TNCS(10°). The Gini index (equation 1) was used as a criterion for calculating discrimination. The decision tree corresponds to the italic entries in Table 3 ▸. (*b*) The confusion matrix.

We used a decision tree (Breiman *et al.*, 1984 ▸), which is a predictive modelling approach used in statistics, data mining and machine learning, to develop criteria for distinguishing between the presence and absence of TNCS (Fig. 4 ▸). The database was divided randomly into a training set (75%) and a test set (25%). The Gini index (equation 1) was used as a criterion for calculating discrimination. The Gini index is a measure of statistical dispersion defined as twice the area between the receiver operating characteristic (ROC) curve and its diagonal:
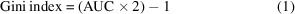



The training set was used to train the algorithm, and included information on pdb-TNCS(*r*°) and the highest non-origin Patterson function peaks. The algorithm resulting from the decision tree was then applied to the test set, which only had the information for the highest non-origin Patterson function peak. Since there was only one parameter to fit for each decision tree (the height of the Patterson function peak) we did not need cross-validation to avoid overfitting. A confusion matrix was generated in order to compute the accuracy (ACC), sensitivity (SN), false-positive rate (FPR) and precision (PREC) of the algorithm, where, given that TP are true positives, TN are true negatives, FP are false positives and FN are false negatives, 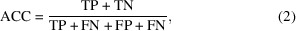


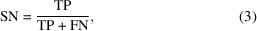


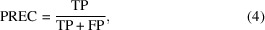


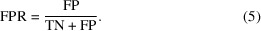



The Patterson function resolution ranges explored were 3–10, 4–10, 5–10, 3–15, 4–15 and 5–15 Å. Following our study of the length of TNCS vectors, only peaks further than 15 Å from the origin peak were accepted.

Tables 3 ▸ and 4 ▸ show that whatever the Patterson function resolution or pdb-TNCS(*r*°) rotational tolerance, suitable Patterson function thresholds based on either percentage of the origin peak or *Z*-scores could be found for high-accuracy decision making; we call the associated threshold *t* values the Patterson-*t*% and Patterson-*Zt*, respectively. Smaller rotational tolerances favoured the use of higher resolution data. Except for five cases highlighted in Table 4 ▸, the Patterson-*Zt* gave slightly higher accuracies than the Patterson-*t*%.

Taking pdb-TNCS(10°) as a useful measure of TNCS, the best predictions, which had 97.6% accuracy (equation 2), used Patterson functions calculated between 5 and 15 Å resolution and a Patterson-*Zt* threshold where *t* = 11.36. Only slightly poorer accuracy, 96.5%, could be obtained using the traditional 5–10 Å resolution range and a Patterson-*t*% threshold, but this required *t* = 16.8% rather than *t* = 20%, implying that the previous Patterson-*t*% threshold for TNCS was too conservative. Since altering the resolution range and using a Patterson-*Zt* threshold had only a marginal effect on accuracy, we decided to use the traditional 5–10 Å resolution range and Patterson-*t*% threshold for our algorithm, although with a lowered threshold value. Using the narrower resolution range also guards against any technical problems when collecting the low-resolution data.

### 3.5. False positives and false negatives   

The false positives and false negatives were further investigated. The sensitivity (equation 3) of the algorithm was 85% and the precision (equation 4) was 88%, while the false-positive rate (equation 5) was 1%, indicating that the algorithm identifies cases of no TNCS exceptionally well, but fails to identify some cases with TNCS. With only one parameter to fit, there is a simple trade-off between identifying false negatives and false positives. The bias in the classifier towards no TNCS comes about because the database contains a higher proportion of structures without TNCS. If we assume that novel data sets will be no more biased towards having TNCS than deposited structures, then the bias is appropriate for accuracy. It is possible that the proportion of crystals that grow with TNCS is higher than that represented by the database because these structures are less likely to be solved; however, we cannot quantify this.

Both false positives and false negatives will impact structure solution by molecular replacement or experimental phasing.

False positives occurred where the top peak in the Patterson function was above the threshold but pdb-TNCS(*r*°) was false. False positives are particularly severe in the context of structure solution because TNCS will be forced to apply to the components in the asymmetric unit (whether molecular-replacement models or heavy atoms) when there is none. Therefore, the false-positive rate (equation 5) of 1% was significant for practical applications even though low.

False negatives occurred where the Patterson function peak was below the threshold proposed by the decision tree but where pdb-TNCS(*r*°) was true. False negatives will mean that intensity modulations are not corrected, and in order to succeed structure solution by molecular replacement will then require high-quality models or, for SAD phasing, the anomalous signal will need to be strong.

Some of the false negatives in the pdb-TNCS(10°) confusion matrix could be rescued by considering a larger angular tolerance. Indeed, 353 of 869 of the false negatives are true according to pdb-TNCS(20°). Note that this is not equivalent to using the decision tree generated with pdb-TNCS(20°), which includes additional false negatives. This phenomenon was true for every pdb-TNCS(*r*°) that we analysed; false negatives could be rescued by considering larger perturbation rotation angles.

### 3.6. TNCS in reciprocal space   

The studies in real space showed that using a Patterson function peak threshold gave high accuracy for detecting TNCS when using pdb-TNCS(*r*°) as the definition of TNCS. However, the optimal Patterson function peak threshold depended critically on the rotation *r* used for the classification, with the Patterson function peak threshold becoming lower as *r* increased. Furthermore, an increasing number of structures that did not have pdb-TNCS(*r*°) were detected as having TNCS as the Patterson function peak threshold was lowered. The studies using the real-space classifier clearly demonstrated the problem of TNCS being a continuum between exact TNCS and NCS. The problem of false negatives lay not in the threshold, but in the real-space classifier of pdb-TNCS(*r*°).

There are several reasons why pdb-TNCS(*r*°) may not correspond to significant modulations in the data. If the TNCS-related components are large, the radius of the molecular G-function (Rossmann & Blow, 1962 ▸) is small so that the modulations fall off faster with orientational differences (Read *et al.*, 2013 ▸). If the TNCS-related copies differ substantially in conformation, the modulations fall off faster with resolution. Finally, if the symmetry-related TNCS vectors are very different, modulations arising from the symmetry-related copies will tend to cancel.

The scope of this study is to determine initial parameters for the model of TNCS so that the refinement of TNCS intensity correction factors can proceed. Therefore, if the resulting modulations are not significant then TNCS is effectively not present for our purposes: if the (insignificant) TNCS epsilon factors are omitted there will be no impact on structure solution.

### 3.7. Epsilon-factor distribution   

**Figure 5:**
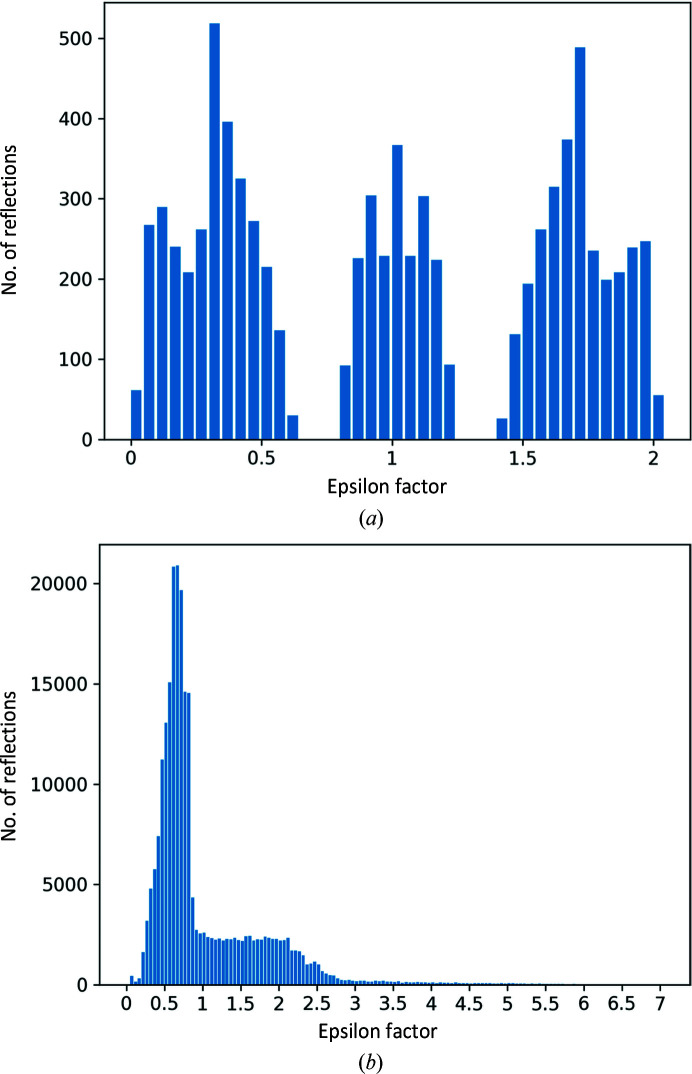
Histograms showing the distribution of refined TNCS epsilon factors for (*a*) PDB entry 2cc0 with σ_1_^2^ = 0.63 for TNCS_2_ (Taylor *et al.*, 2006 ▸) and (*b*) PDB entry 4n3e with σ_1_^2^ = 0.61 for TNCS_7_ (Sliwiak *et al.*, 2014 ▸).

We examined the distribution of epsilon factors after refinement as an alternative classifier for the presence or absence of TNCS. Refined epsilon factors that cluster around 1 define unmodulated data, while those that refine to the extremes of the distribution define high modulation. We use the variance about 1 (σ_1_^2^) as the statistic for measuring the degree of modulation,
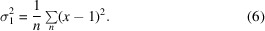



We call this eps-TNCS, and it takes a range of values between 0 and (*n*/2)^2^ + [(*n*/2) − 1]^2^, although in practice it is less than 1 in all but extraordinary circumstances. Histograms showing examples of the distribution of epsilon factors and their associated eps-TNCS are presented in Fig. 5 ▸.

**Figure 6:**
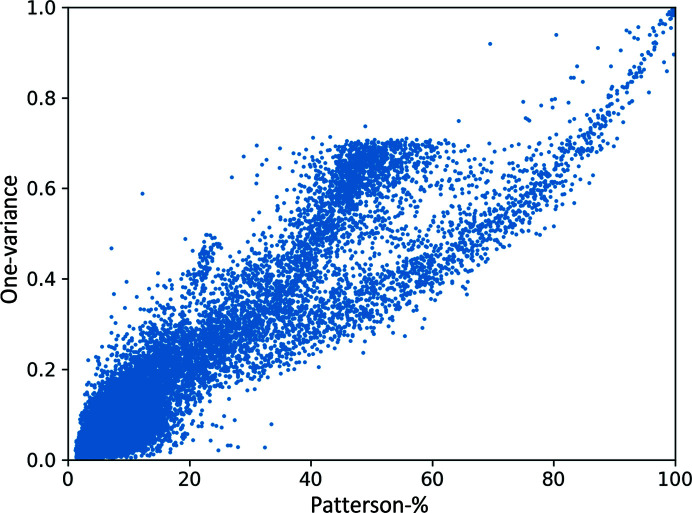
Scatter plot showing the distribution of refined TNCS epsilon factor one-variance (variance about 1; equation 6) for all cases with pdb-TNCS(20°). Data resolution range 5–10 Å.

The distribution of eps-TNCS values versus Patterson-*t*% is shown in Fig. 6 ▸. There is a clear linear relationship between the two: Patterson peak height is directly related to modulation in the data. The Patterson-*Zt* had a lower correlation coefficient (0.82) with the eps-TNCS than the Patterson-*t*%. The correlation coefficient between the eps-TNCS and the Patterson-*t*% was 0.934 and was calculated with eps-TNCS refined against 5–10 Å resolution data and Patterson functions calculated with 5–10 Å resolution data.

This analysis demonstrated that the false negatives in the algorithm, as determined by pdb-TNCS(*r*°) (a binary measure), were cases in which the eps-TNCS (a real number) was low and therefore their misclassification should not strongly impact structure solution. It also demonstrates that the Patterson function peak height is a good measure for the ranking of a TNCS hypothesis.

### 3.8. Completeness   

It has long been known that complete, good-quality data are necessary for successful molecular replacement using Patterson function methods (Navaza, 1994 ▸). In the course of our study, we noted that the completeness of the data had a significant effect on the accuracy of our Patterson function-based decision tree. Eight cases [PDB entries 3c6o (Hayashi *et al.*, 2008 ▸), 1jpn (Padmanabhan & Freymann, 2001 ▸), 1sxh (Schumacher *et al.*, 2004 ▸), 1n8o (C. Cambillau, S. Spinelli & M. Lauwereys, unpublished work), 1eam (Hu *et al.*, 1999 ▸), 1wwr (Kuratani *et al.*, 2005 ▸), 3it5 (Spencer *et al.*, 2010 ▸) and 1lbs (Uppenberg *et al.*, 1995 ▸)] had high Patterson function peaks but no significant epsilon-factor dispersion. There was one outlier (PDB entry 3he1; Osipiuk *et al.*, 2011 ▸) with a variance about 1 (equation 6) of nearly 1.6 for TNCS_6_, the only case we observed for which σ_1_^2^ was greater than one (Supplementary Fig. S2). This figure shows that low-completeness data resulted in several other outliers in the Patterson-*t*% versus σ_1_^2^ scatter plot. The accuracy of the decision tree deteriorated with decreasing completeness (Supplementary Fig. S3). We have not investigated the distribution of missing data in these data sets; however, when large percentages of data are missing it is normally because the user has failed to collect a wedge of data, either through initial misidentification of the true space group, radiation damage causing data quality to drop so that later parts of a data collection must be excluded, or a high number of overlapped reflections in a section of the data (for example due to one long unit-cell dimension). Lacking a wedge of data will impact the eps-TNCS refinement because systematic omission of data for a direction in reciprocal space leaves parameters in real space perpendicular to that direction undefined. In addition, missing wedges of data complicate data processing, and if due to overlaps some reflections may be integrated including partial intensity from a neighbouring reflection; any such rogue high-intensity reflections cause strong modulation of the Patterson function.

### 3.9. Lattice-translocation disorder   

For the cases of false positives, Patterson functions were calculated from the coordinates and compared with the observed Patterson functions. In all cases, the highest non-origin Patterson function peak from the calculated data was below the 20% threshold. It is possible that these structures show a degree of lattice-translocation disorder, with stacking heterogeneity between mosaic blocks (Rye *et al.*, 2007 ▸; Dauter *et al.*, 2005 ▸). Interestingly, the distribution of space groups in these structures differed significantly from the distribution across all deposited structures, with space group *P*2_1_ present at three times the expected number (see Table 5 ▸). The 2_1_ screw has been implicated as an important component of polytropism for crystals (Aquilano *et al.*, 2003 ▸).

## 4. TNCS detection   

Our algorithm for TNCS detection not only determines the TNCS vector and the TNCS order, but also has tests that aim to exclude pathological cases. Firstly, a Patterson function is calculated from the data, by default using 5–10 Å resolution data. Peaks are picked in the Patterson function and filtered using two criteria: the peak height must be above a given percentage of the origin-peak height and the peak distance must be more than a given distance from the origin. As guided by this study, the default distance threshold is 15 Å and the default Patterson function threshold is 16.8%. Cases in which at least one of the unit-cell dimensions is less than the origin distance threshold are considered to be pathological (most likely peptides) and are excluded from further analysis. If there are no surviving non-origin distinct peaks over the Patterson-% threshold, the algorithm terminates with status ‘TNCS not indicated’, otherwise the algorithm proceeds to analysis of the TNCS order. The simplest interpretation of surviving peaks is that each (if there are more than one) presents an independent TNCS_2_ vector, with Patterson-% indicating the strength of the associated modulation, which provides a ranking for the hypotheses.

We then perform further analysis to determine whether the Patterson function peaks are due to a higher order TNCS commensurate modulation and, if so, the order of that commensurate modulation. Noise in the Patterson function is removed by setting all values below 8% of the Patterson function origin peak to zero, and the noise-reduced Patterson function is transformed to reciprocal space, where commensurate modulation is detected as strong low-order Fourier terms. The hypothesis for a given commensurate modulation will predict a set of equal-height peaks in the Patterson function. In practice, because the components are not related by a perfect translation (as previously discussed) these predicted peaks will have different heights and some may be below the Patterson-*t*% threshold of the analysis. Following our studies on eps-TNCS and the high correlation with the height of the highest Patterson function peak, we rank commensurate modulations that predict the highest ranked peak higher than those that do not.

The result of the algorithm is a ranked list of TNCS modulations representing high-order commensurate TNCS*_n_* and commensurate and non-commensurate TNCS_2_. Following our observation that high Patterson function peaks in the data may be due to order–disorder effects, the case of no TNCS is also always included in the list of hypotheses. Note that the ranking is not necessary for structure solution. In the context of an automated pipeline, as long as the correct hypothesis is in the list, it will be explored. The ranking only affects the order in which the hypotheses are explored, and hence the efficiency of structure solution.

An unoptimized part of the algorithm attempts to prevent the misclassification of coiled coils and amyloid peptide repeats as having TNCS. As previously discussed, pseudo-symmetry in secondary-structure elements generates large peaks in the Patterson function close to the origin. Although coiled coils were excluded from our curated database, by looking at a small number of cases it was observed that the 15 Å minimum vector exclusion around the origin was not sufficient to exclude peaks generated by the coiled-coil pseudo-symmetry (Kondo *et al.*, 2008 ▸). Taking a heuristic approach, we exclude peaks from the TNCS analysis if they cluster together with the short distance separation characteristic of coiled coils. Future work will perform a systematic study of coiled coils and amyloid peptide repeats to optimize the TNCS-detection algorithm in these cases. Note that it is the clustering of a number of Patterson function peaks corresponding to the helical repeat distance that is characteristic of coiled coils, rather than the presence of a peak close to the origin *per se*.

## 5. Discussion   

We have developed an algorithm for characterizing and ranking TNCS hypotheses by analysis of the intensities prior to structure solution. Correct identification of TNCS can have a profound impact on the ability to place components in the asymmetric unit, whether they be components by molecular replacement or heavy atoms by experimental phasing. In the context of a pipeline for structure solution, the fastest route to structure solution on average should be by exploring the TNCS hypotheses in order of ranking by our criteria. Future work will develop our automation strategies to make optimal use of this information and will include dynamic re-ranking of TNCS hypotheses.

Unexpectedly, several entries in our database had significant Patterson function peaks despite not having TNCS. One of these cases was the proteolytic domain of *Archaeoglobus fulgidus* Lon protease (Dauter *et al.*, 2005 ▸; PDB entry 1z0v), a structure known to be an allotwin (see also PDB entry 1z0t; Lebedev, 2009 ▸). Individual crystals belonged to space groups *P*2_1_ and *P*2_1_2_1_2_1_, with the transition layers in plane space group *P*2_1_2_1_(2) giving a sequence of stacking vectors. Another case was lipase B from *Candida antarctica*, which is also known to be an order–disorder twin. In this case, the two space groups involved were *C*2 and *P*2_1_2_1_2_1_, with the transition layers again in plane space group *P*2_1_2_1_(2). The deposited data for PDB entry 1lbs (Uppenberg *et al.*, 1995 ▸) were processed in the larger, orthorhombic lattice, which resulted in an apparent data completeness of 27.5%, although the completeness in the actual *C*2 space group was 82.4%. In terms of our study, this structure was included in the small database of structures with less than 80% complete data; however, had it been included in the main database it would have been the most extreme false-positive outlier. In another case, the Ftsk motor domain from *Escherichia coli* (Massey *et al.*, 2006 ▸; PDB entry 2ius), the indexing and space-group determination for the crystal was problematic (Jan Löwe, personal communication). We thus hypothesize that these outliers are as a result of structures with a lattice-translocation defect rather than TNCS. In the context of automated structure determination, it is therefore important to consider the absence of TNCS even in the context of large Patterson function peaks being present.

In the course of our study, we also noted a few cases in which subgroups of components were related by different TNCS vectors. These cases tended towards pseudo-centring in multiple directions. For example, a small ligand-bound complex of von Hippel–Lindau (VHL) E3 ubiquitin ligase and the hypoxia-inducible factor (HIF) alpha subunit (Galdeano *et al.*, 2014 ▸; PDB entry 4w9d; *P*4_1_22) showed a pseudo-centring in the *a* (0.5, 0.04, 0.0) and *ab* diagonal (0.54, 0.5, 0.0) directions, and similarly the crystal structure of the SOAR domain (Yang *et al.*, 2012 ▸; PDB entry 3teq; *P*4_1_2_1_2) showed pseudo-centring in the *a* (0.49, 0.01, 0.0) and *ab* diagonal (0.49, 0.51, 0.0) directions. If there are subgroups of components related by different TNCS vectors or if only some components of the asymmetric unit are related by a TNCS vector, then the modulations of the expected intensities due to the TNCS will be much less significant and structure solution may be achieved without any TNCS correction being applied, as indeed was the case in these examples. However, if structure solution fails, detecting and correcting the dominant order of TNCS within the asymmetric unit may be enough.

In this work, we have not attempted to model either the TNCS rotation or the TNCS r.m.s.d. from the Patterson function. Some information about these parameters is contained in the Patterson function peak height relative to the origin peak, with lower peak heights indicating greater deviation from perfect translation. There may also be information about rotational deviations in the three-dimensional Patterson function peak shape. However, in practice, refinement of these parameters from several different TNCS rotation perturbations works extremely well, and in most cases all perturbations converge on refinement to the same final TNCS rotation and TNCS r.m.s.d.

Future improvements to the method could come from improvements in the coefficients used to calculate the Patterson function. Down-weighting coefficients with high experimental error may mitigate the differences seen between Patterson functions calculated with different resolution ranges. Work is in progress to optimize the information in Patterson-like functions in this, and other, crystallographic contexts.

## Supplementary Material

Supplementary Figures. DOI: 10.1107/S2059798320016836/gm5078sup1.pdf

## Figures and Tables

**Table 1:** Summary of database curation

Initial database	90083	(substituted)
Obsolete PDB files	−296	
Substituted by data from PDB_REDO		357
Failure of our scripts and not in PDB_REDO or still error	−331	
MTRIX	−2	15
SCALE		16
Structures refined as ensembles	−79	
Disordered structures, mean occupancy < 0.75	−92	
C^α^-only structures	−21	
Contains nucleic acids	−5445	
Highly helical structures (coiled coils, transmembrane proteins…)	−1712	
Collagen	−32	
Viruses	−202	
Antibiotics	−36	
Peptides	−59	
Data completeness below 80%	−1294	
Final database	80482	

**Table 2:** Results of the coordinate analysis depending on different rotational tolerance ranges (accumulative) The results show the number of structures with TNCS and the percentage of the total database, the number of structures with two molecules related by TNCS and the number with more than two molecules related by TNCS.

Rotational tolerance	TNCS	TNCS order = 2	TNCS order > 2
0–2°	2523 (3.13%)	2375	148
0–5°	4818 (6.00%)	4332	486
0–10°	7503 (9.30%)	6660	843
0–15°	9549 (11.86%)	8396	1153
0–20°	11230 (13.95%)	9822	1408

**Table 3:** Accuracy (percentage) of the decision trees and the best value of Patterson-*Zt* depending on the rotational tolerance and resolution ranges used for calculation of the Patterson The values in italics have the highest accuracy for pdb-TNCS(10°) and are discussed in the text (Fig. 4 ▸).

	0–2°		0–5°		0–10°		0–15°		0–20°	
	Accuracy	*Z*-score	Accuracy	*Z*-score	Accuracy	*Z*-score	Accuracy	*Z*-score	Accuracy	*Z*-score
3–10 Å	98.10	46.81	98.23	28.90	96.97	12.81	94.96	9.80	93.10	9.80
4–10 Å	97.68	33.70	98.19	20.33	97.17	11.49	95.14	10.35	93.20	9.60
5–10 Å	97.22	24.97	97.94	16.51	97.36	10.82	95.29	9.35	93.36	8.65
3–15 Å	98.03	46.91	98.23	28.82	97.07	12.86	95.31	10.09	93.28	9.57
4–15 Å	97.67	36.00	98.09	21.04	97.26	10.84	95.45	9.63	93.47	9.60
5–15 Å	97.02	26.39	97.74	17.90	*97.59*	*11.36*	95.63	9.66	93.83	9.06

**Table 4:** Accuracy (percentage) of the decision trees and the best value of Patterson-*t*% depending on the rotational tolerance and resolution ranges used for calculation of the Patterson The values in bold are higher than the corresponding values in Table 3 ▸. The values in italics are discussed in the text.

	0–2°		0–5°		0–10°		0–15°		0–20°	
	Accuracy	Percentage	Accuracy	Percentage	Accuracy	Percentage	Accuracy	Percentage	Accuracy	Percentage
3–10 Å	97.95	28.13	97.66	15.83	95.99	8.31	94.05	7.63	91.97	8.31
4–10 Å	**97.75**	32.38	97.59	18.17	96.21	11.86	94.17	11.70	92.05	11.59
5–10 Å	**97.34**	34.39	97.37	19.85	*96.46*	*16.80*	94.39	15.40	92.31	15.53
3–15 Å	**98.04**	30.48	97.65	15.52	96.16	8.31	94.36	7.52	92.21	7.55
4–15 Å	**97.79**	34.20	97.56	18.67	96.39	11.62	94.43	10.71	92.30	10.73
5–15 Å	**97.22**	36.25	97.24	19.24	96.61	16.41	94.70	15.56	92.64	15.52

**Table 5:** Space-group propensity for 158 cases where there was a high peak in the Patterson function but no TNCS in the coordinates PDB average is given as a percentage following Wukovitz & Yeates (1995 ▸).

	No.	Percentage	PDB average (%)
*P*2_1_	60	38	11.1
*C*2	30	19	6.1
*P*1	23	15	2.6
*P*2_1_2_1_2_1_	8	5	36.1
*P*2_1_2_1_2	5	3	3.7
*C*222_1_	5	3	3.7
*H*32	5	3	—
*H*3	5	3	—
