# Supplementary material for: Detection of translational noncrystallographic symmetry in Patterson functions
Source: Acta Crystallogr D Struct Biol. 2021 Feb 5;77(Pt 2):131–41. doi: 10.1107/S2059798320016836 (PMC7869901; doi:10.1107/S2059798320016836)

STRUCTURAL BIOLOGY

Volume 77 (2021)

Supporting information for article:

Detection of translational noncrystallographic symmetry in Patterson functions

Iracema Caballero, Massimo D. Sammito, Pavel V. Afonine, Isabel Usón, Randy J. Read and Airlie J. McCoy

Figure S1 Non-cumulative histograms of the number of structures with a highest non origin peak value, depending on the rotational tolerances and the resolution used for calculating the Patterson function map. a) Patterson function peak percentages for structures with TNCS are shown, with a red line drawn at 20% b) Patterson function peak Z-scores for structures with TNCS. c) Patterson function peak percentages for structures without TNCS. d) Patterson function peak Z-scores for structures without TNCS. Panels a) and b) illustrate the decrease in modulation for structures with TNCS as rotational tolerance increases for all the resolution ranges used for calculating the Patterson function map. Panels c) and d) shows that the majority of the structures without TNCS have a low Patterson function peak.

a)

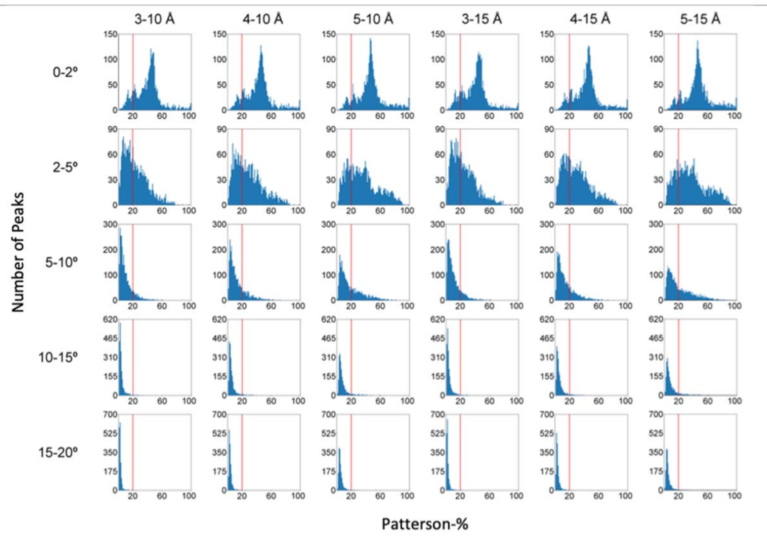

b)

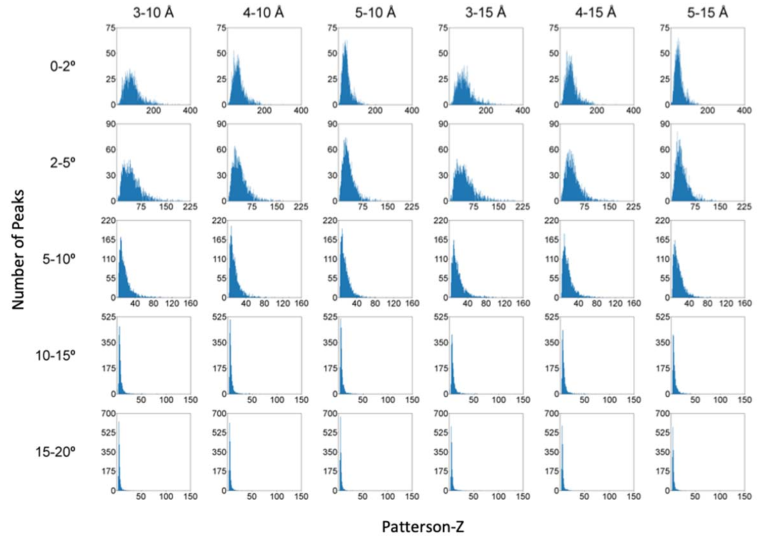

c)

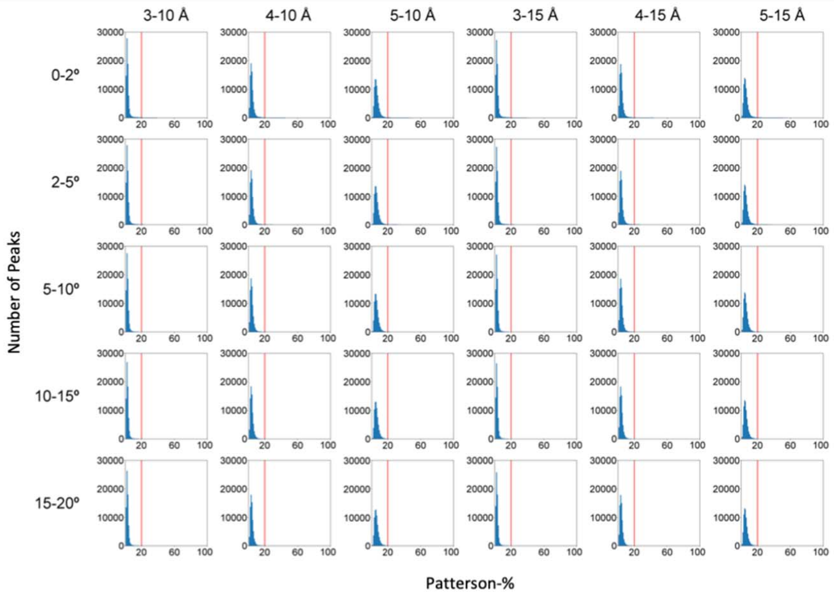
3-10 Å
4-10 Å
5-10 Å
3-15 Å
4-15 Å
5-15 Å
0-2º
2-5º
5-10º
10-15º
15-20º
Number of Peaks
Patterson-%

d)

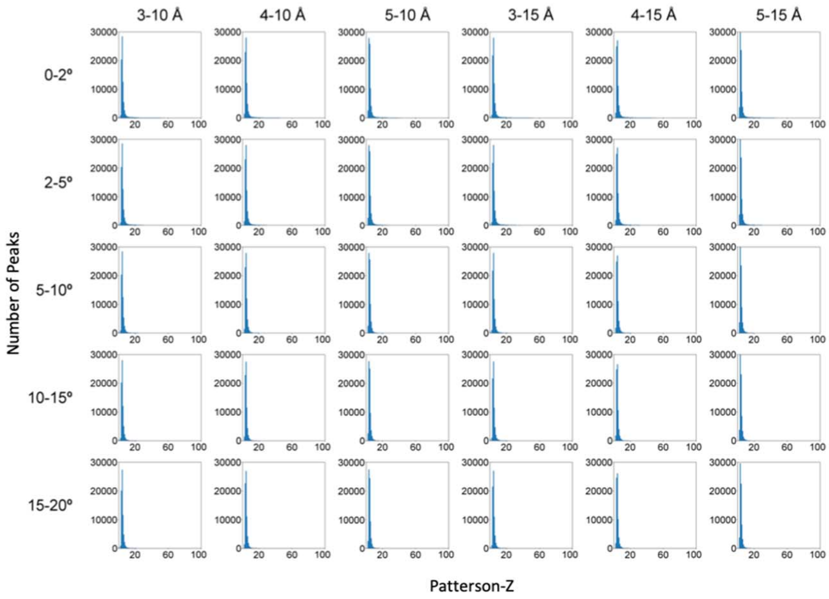
3-10 Å
4-10 Å
5-10 Å
3-15 Å
4-15 Å
5-15 Å
0-2º
2-5º
5-10º
10-15º
15-20º
Number of Peaks
Patterson-Z

Figure S2 Scatter plot showing the distribution of refined TNCS epsilon factor variances for all cases with pdb-TNCS(20°) and data completeness less than 80%, which were excluded from our database.

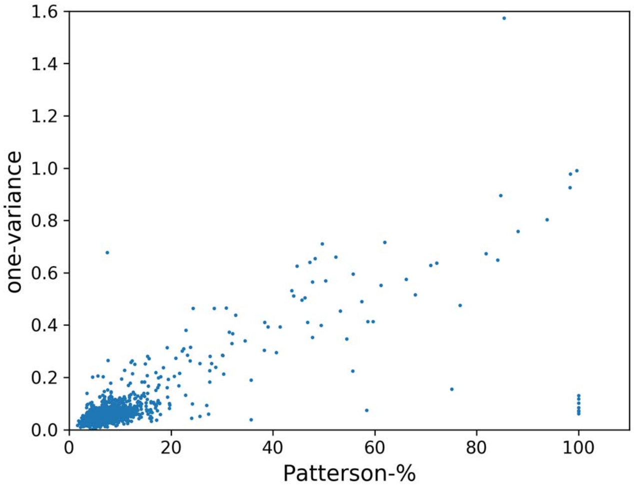

Figure S3 Histogram showing how the completeness of the data affects the accuracy of the decision tree. Low data completeness causes the algorithm to become much less reliable

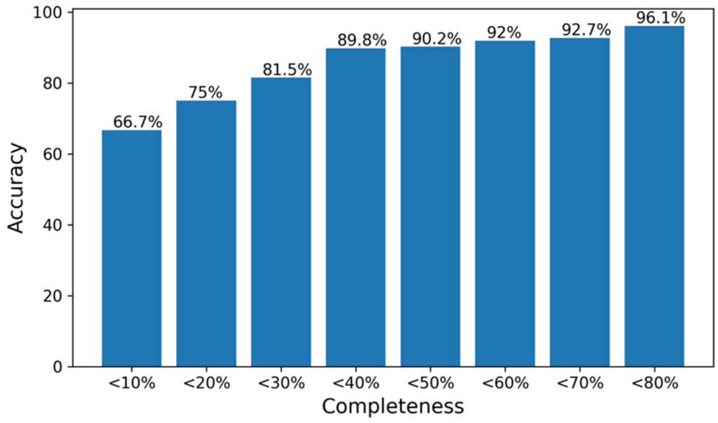

Supplement: Supplementary file 1 [file d-77-00131-sup1.pdf]
